# A biphasic growth model for cell pole elongation in mycobacteria

**DOI:** 10.1038/s41467-019-14088-z

**Published:** 2020-01-23

**Authors:** Mélanie T. M. Hannebelle, Joëlle X. Y. Ven, Chiara Toniolo, Haig A. Eskandarian, Gaëlle Vuaridel-Thurre, John D. McKinney, Georg E. Fantner

**Affiliations:** 1 School of Engineering, Swiss Federal Institute of Technology (EPFL), 1015 Lausanne, Switzerland; 2 School of Life Sciences, Swiss Federal Institute of Technology (EPFL), 1015 Lausanne, Switzerland

**Keywords:** Atomic force microscopy, Cell growth, Cellular microbiology, Pathogens

## Abstract

Mycobacteria grow by inserting new cell wall material in discrete zones at the cell poles. This pattern implies that polar growth zones must be assembled de novo at each division, but the mechanisms that control the initiation of new pole growth are unknown. Here, we combine time-lapse optical and atomic force microscopy to measure single-cell pole growth in mycobacteria with nanometer-scale precision. We show that single-cell growth is biphasic due to a lag phase of variable duration before the new pole transitions from slow to fast growth. This transition and cell division are independent events. The difference between the lag and interdivision times determines the degree of single-cell growth asymmetry, which is high in fast-growing species and low in slow-growing species. We propose a biphasic growth model that is distinct from previous unipolar and bipolar models and resembles “new end take off” (NETO) dynamics of polar growth in fission yeast.

## Abstract (web summary)

Mycobacteria grow by inserting new cell wall material at the cell poles. Here, Hannebelle et al. combine time-lapse optical and atomic force microscopy to show that single-cell growth is biphasic due to a lag phase of variable duration before the new pole transitions from slow to fast growth.

## Introduction

At the single-cell level, the spatial pattern of cell growth and division is remarkably diverse, even among organisms that are morphologically similar^1^. Among rod-shaped bacteria, some species grow by insertion of new material into the sidewalls with little or no growth at the poles (e.g., *Escherichia coli, Bacillus subtilis*)^1^, while others show the opposite pattern, growing by insertion of new material at the poles with little or no growth at the sidewalls (e.g., *Mycobacterium* species)^1^. *Mycobacterium* is a medically relevant genus that includes important pathogenic species such as *M. tuberculosis* and *M. leprae*.

To characterize the pattern of growth of a bacterium at the single-cell level, both temporal and spatial information are required with sufficiently high resolution. Several methods have been developed over the past decades for measuring the pattern of single-cell growth, but the conclusions have varied between studies. Two main models have been proposed for bacteria that grow by extension of the sidewalls, such as *E. coli* or *B. subtilis*: a *linear model* in which cells grow at a constant speed^2^, and an *exponential model* in which the speed of growth is proportional to cell size^3^. More recently, the exponential growth pattern has been confirmed for *B. subtilis* by a pioneering study using a suspended microchannel resonator to measure the buoyant mass of individual cells over time^4^.

**Fig. 1:**
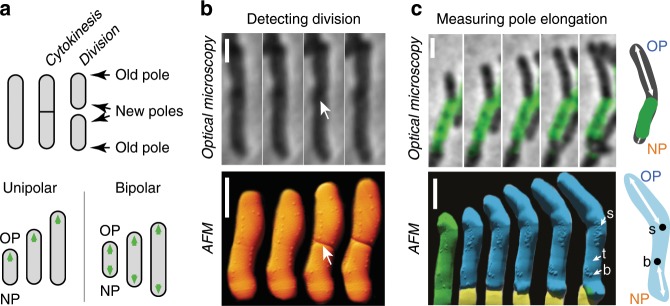
Measurement of *M. smegmatis* pole elongation dynamics using AFM. **a** Schematic of unipolar^8^ and bipolar^7^ elongation models. OP, old pole. NP, new pole. **b** Comparison between phase-contrast and AFM time-lapse images of dividing cells measured on different instruments. Arrows indicate the division site in the first frame following the division event. For phase-contrast microscopy data, the division event was detected using the method described in Supplementary Fig. 5. Scale bar, 1 μm. Time between consecutive images is 10 min for phase-contrast microscopy data and 13 min on average for AFM data. **c** Absolute measurement of pole elongation using fluorescence pulse-chase labeling and AFM-resolved surface nanostructures as fiducial markers. Scale bar, 1 μm. Top: combined phase-contrast and fluorescence images of an elongating cell. Time between consecutive images is 30 min. The schematic illustrates how fluorescently labeled cell wall (green) can be used as a fiducial marker to measure pole elongation (white arrows). Bottom: AFM time-lapse images of a mother cell (green) and its two daughter cells (blue and yellow). Surface nanostructures used as fiducial markers are indicated with a white arrow: division scar (s), protruding bleb (b), trough (t). The schematic illustrates how surface nanostructures (●) are used as fiducial markers to measure pole elongation over time. Time between two images is 1.25 h on average.

Compared to our understanding of the growth pattern of sidewall-growing organisms, our understanding of polar growth is incomplete^5^. Time-lapse optical microscopy combined with microfluidics has become a tool of choice for measuring the pattern of polar growth in mycobacteria^6–10^. Despite a consensus that mycobacteria grow exclusively at the poles, it remains controversial whether their pattern of single-cell growth follows unipolar (asymmetric) or bipolar (symmetric) dynamics^5,7–11^. According to the unipolar model, the new cell pole grows very slowly or not at all between birth and division, when it becomes the old pole of the newborn cell and transitions to fast growth (Fig. 1a). According to the bipolar model, both poles, new and old, grow at the same rate between birth and division (Fig. 1a).

We sought to reexamine this controversy regarding the pattern of single-cell growth in mycobacteria using time-lapse microscopy with high spatial resolution. The spatial resolution of optical microscopy is limited by diffraction to approximately half the wavelength of light, which corresponds in size to the radius of a bacterium. Super-resolution optical microscopy can overcome the diffraction limit^12–14^, but most super-resolution techniques are not compatible with long-term time-lapse imaging due to phototoxicity^15^. Atomic force microscopy (AFM) is emerging as a powerful tool for microbiology, as it allows nanometer resolution imaging of live cells in liquid cultures^16,17^. AFM has been successfully used to study cell wall nanostructure^18–20^, cell growth^18,19^, and the nanoscale effects induced by drug exposure^20,21^. In addition, developments in AFM technology have enabled imaging of bacterial processes at high temporal resolution^22–24^. Recently, we developed methods for long-term time-lapse AFM to observe mycobacteria growing and dividing through multiple generations with nanometer resolution^25^. AFM time-lapse images revealed morphological landmarks on the mycobacterial cell surface, which appear up to two generations in advance and correspond to future sites of division^25^. Using AFM nanomechanical mapping, we found that mycobacterial division is driven by a combination of peptidoglycan hydrolytic activity and accumulation of mechanical stress at the septum, which culminates in abrupt division in a timeframe of milliseconds^26^.

Here, we use a combination of time-lapse AFM and optical microscopy to characterize the spatial and temporal pattern of single-cell growth in fast- and slow-growing *Mycobacterium* species. We show that single-cell growth of mycobacteria follows biphasic polar dynamics distinct from the previously proposed unipolar and bipolar models.

## Results

### Measurement of pole elongation with time-lapse AFM

We imaged cell growth and division of *Mycobacterium smegmatis* with time-lapse AFM, which provides nanometer resolution three-dimensional topographical data of the cell surface (Supplementary Movie 1). Cell division is visible in the AFM images as a sudden drop in height at the nascent division site^25,26^. Upon cell division, two newly formed sibling cells often remain closely apposed at their new poles and may therefore appear to be a single non-divided cell by optical microscopy while being resolvable as two distinct cells by AFM (Fig. 1b). Moreover, AFM reveals nanoscale structures on the cell surface, such as wave-troughs^25^, division scars^27^, and protruding blebs, which may represent microvesicles^28^ (Fig. 1c; Supplementary Fig. 1). These surface structures are spatially immobile over time because they are outside the polar growth zones (Supplementary Fig. 1), which validates them as fiducial markers that can be used to measure the growth of each cell pole individually. By repeatedly measuring the distance between cell surface fiducial markers and the cell poles, we were able to quantify pole elongation in absolute terms (Fig. 1c; Supplementary Fig. 2) with ∼50 nm precision (Supplementary Fig. 3).

### Pole elongation follows “new end take off” (NETO) dynamics

**Fig. 2:**
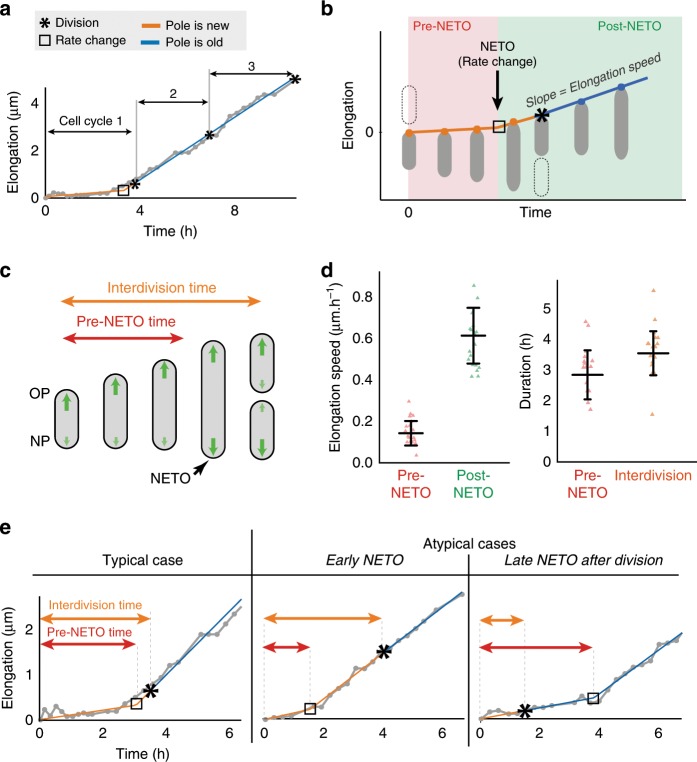
Biphasic pole growth: NETO and cell division are independent events. **a** Elongation of a representative newborn pole through three generations measured by time-lapse AFM. Gray: AFM data; orange/blue: bilinear fit. See Supplementary Fig. 4. **b** Schematic of biphasic growth of a newborn pole with a rate change (NETO). **c** Schematic of biphasic NETO growth of a cell over one cell cycle. **d** Left: elongation speeds of newborn poles during the pre-NETO phase (slow or no growth) and the post-NETO phase (fast growth). Right: pre-NETO time and interdivision time. Data from time-lapse AFM images (20 poles). Bars indicate averages and standard deviations. **e** Typical elongation curve of a newborn cell pole (left) and atypical curves with early NETO before division (center) and late NETO after division (right). Each curve corresponds to one of the data points in **d**. Gray: AFM data; orange/blue: bilinear fit.

We observed that a newly born cell pole initially grows slowly (or not at all) before transitioning to a constant rate of fast growth (Fig. 2a), reminiscent of the unipolar model^8^. However, close inspection of our AFM data reveals that, in most cases, the new pole transitions from slow to fast growth prior to the subsequent cell division (Fig. 2a). Rather than unipolar^8,10^ or bipolar^7^ growth, this pattern (Fig. 2b; Supplementary Fig. 4) instead resembles the NETO model of biphasic pole growth described previously in the eukaryotic fission yeast *Schizosaccharomyces pombe*^29^. Although mycobacteria and fission yeast are separated by more than a billion years of evolution, we will use the descriptive term “NETO” in reference to the dynamics of polar growth in mycobacteria as well (Fig. 2c).

**Fig. 3:**
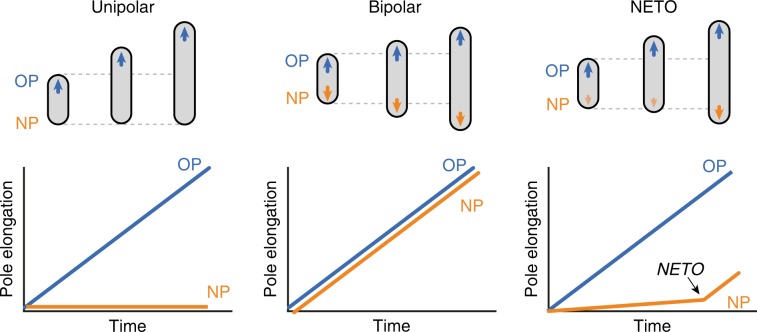
Unipolar, bipolar, and NETO pole growth models in mycobacteria. Schematic representation of the elongation curves for the old pole (OP, blue) and the new pole (NP, orange) of a cell from birth (division of the mother cell) to division, according to the unipolar^2^, bipolar^3^, and NETO models.

We found that the lag phase between new pole formation and the transition from slow growth (average ~0.15 ± 0.06 µm per hour) to fast growth (~0.61 ± 0.13 µm per hour) is variable with an average duration of 2.9 ± 0.8 h in cells growing with an average interdivision time of 3.6 ± 0.7 h (Fig. 2d). In the majority (>80%) of cells, NETO occurs between birth and the next cell division (Fig. 2e). However, in a minority (<20%) of cells, NETO occurs after the next cell division (Fig. 2e), which rules out the possibility that division *per se* is the trigger that causes a cell pole to switch from slow to fast growth. We conclude that NETO and cell division are temporally distinct events in terms of their relative order and timing. The NETO pattern of growth is distinct from the bipolar growth model^7^, according to which fast pole growth is initiated immediately at pole birth (Fig. 3). The NETO pattern of growth is also distinct from the unipolar growth model^8^, according to which fast pole growth is initiated at cell division, when the new pole becomes an old pole (Fig. 3).

We investigated whether NETO growth dynamics might be an artifact caused by the experimental conditions we used for time-lapse AFM. As a comparison, we analyzed data obtained by phase-contrast microscopy of bacteria growing in a microfluidic device using the same experimental conditions under which bipolar growth was previously reported^7^. In this device, the cells are sandwiched between a glass coverslip and a semipermeable membrane, which constrains them to grow in the imaging plane while providing a continuous supply of nutrients. Separation of newly divided sibling cells can be difficult to observe in diffraction-limited phase-contrast microscopy images. However, time-lapse AFM images revealed that division was accompanied by an abrupt snapping movement of the sibling cells away from each other by ~100 nm on average (Supplementary Fig. 5a). Based on the insights provided by our AFM experiments, we developed an algorithm using differential image processing to identify the snapping separation of sibling cells in phase-contrast microscopy images by measuring the differences between consecutive frames (Supplementary Fig. 5b). We then used morphological features that were visible by phase-contrast microscopy on a minority of cells (e.g., bent cells, constrictions) as low-precision fiducial markers to measure the elongation of individual cell poles (Supplementary Fig. 5c). Our results with phase-contrast microscopy confirmed that newborn cell poles grow with biphasic NETO dynamics, in agreement with the results we obtained in AFM time-lapse experiments (Supplementary Fig. 5d). We conclude that NETO growth dynamics are not an artifact caused by the experimental conditions that we use for time-lapse AFM imaging. Moreover, our AFM time-lapse data allowed us to validate a new and more accurate method to measure pole elongation from birth to division using diffraction-limited optical time-lapse images.

### NETO is not due to release of physical constraints on poles

**Fig. 4:**
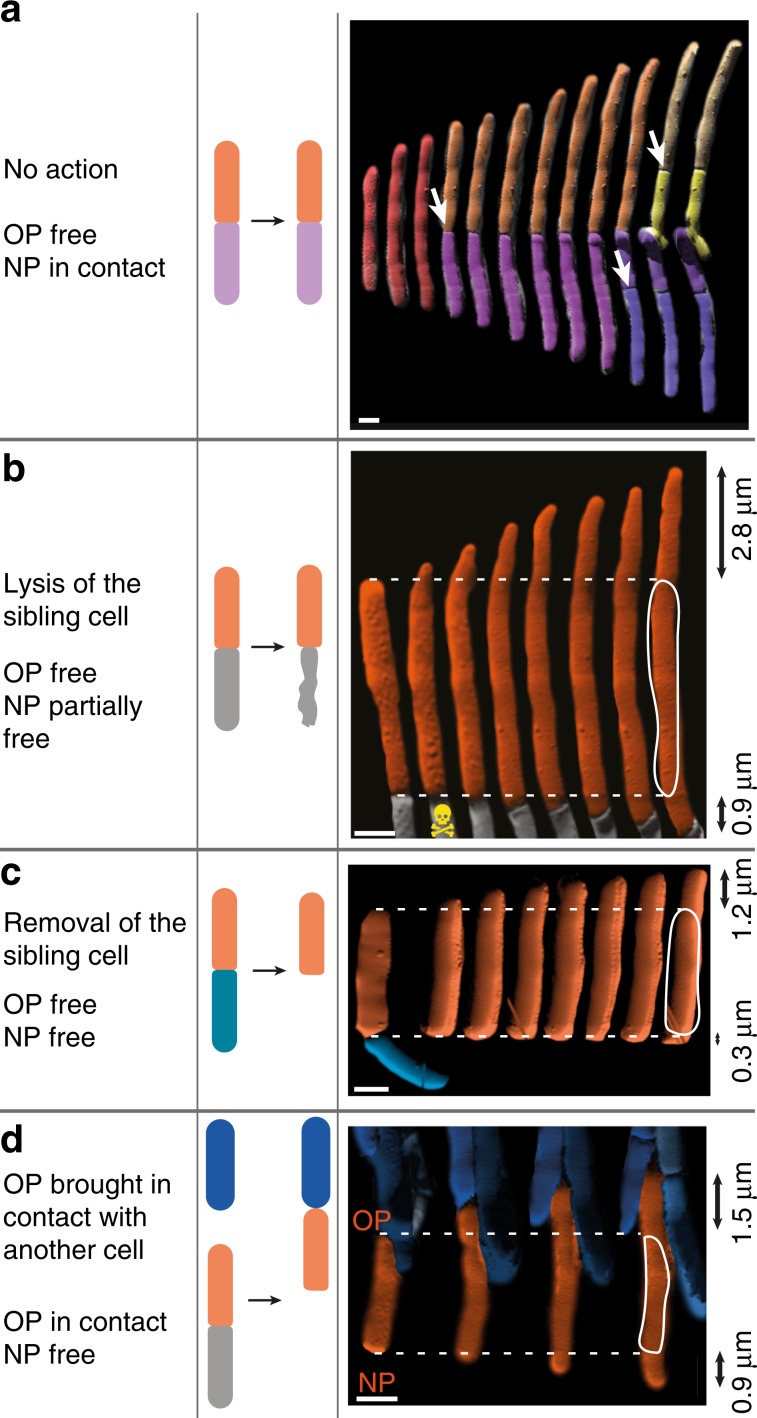
The pre-NETO phase is not due to physical constraints on new cell poles. **a**–**d** Schematics and corresponding time-lapse AFM of growing *M. smegmatis* cells. Black arrows indicate the cumulated elongation of the old pole (OP) and the new pole (NP). Scale bar, 1 μm. **a** Close contact of new poles of newborn sibling cells is maintained after division of the mother cell (white arrows). **b** Elongation of cell poles after the new pole was partially freed by ablating the turgor pressure in one of the sibling cells using the sharp AFM tip (skull). **c** Elongation of cell poles after the new pole was freed by removal of one of the sibling cells using the AFM cantilever. **d** Elongation of cell poles after the new pole was freed and the old pole was physically obstructed by moving it into contact with other cells using the AFM cantilever.

We hypothesized that the lag phase between birth and NETO might result from spatial constraint inhibiting growth of the new cell pole, since newborn cells remain in physical contact for some time after cell division (Fig. 4a). We relieved this spatial constraint by using the AFM cantilever tip to mechanically lyse or physically displace one cell in a pair of siblings (Supplementary Fig. 6). Neither of these manipulations eliminated the lag phase between new pole birth and NETO (Fig. 4b, c). Conversely, inverting the orientation of a cell in order to bring its old pole into physical contact with another cell pole (Supplementary Fig. 6) had no impact on the growth dynamics of the old pole, which continued to elongate at the same rate (Fig. 4d). We conclude that the spatial constraint imposed by end-to-end physical contact between newborn sibling cells is not responsible for the slow rate of new pole elongation during the pre-NETO phase.

### Delayed accumulation of Wag31 at the new cell pole

**Fig. 5:**
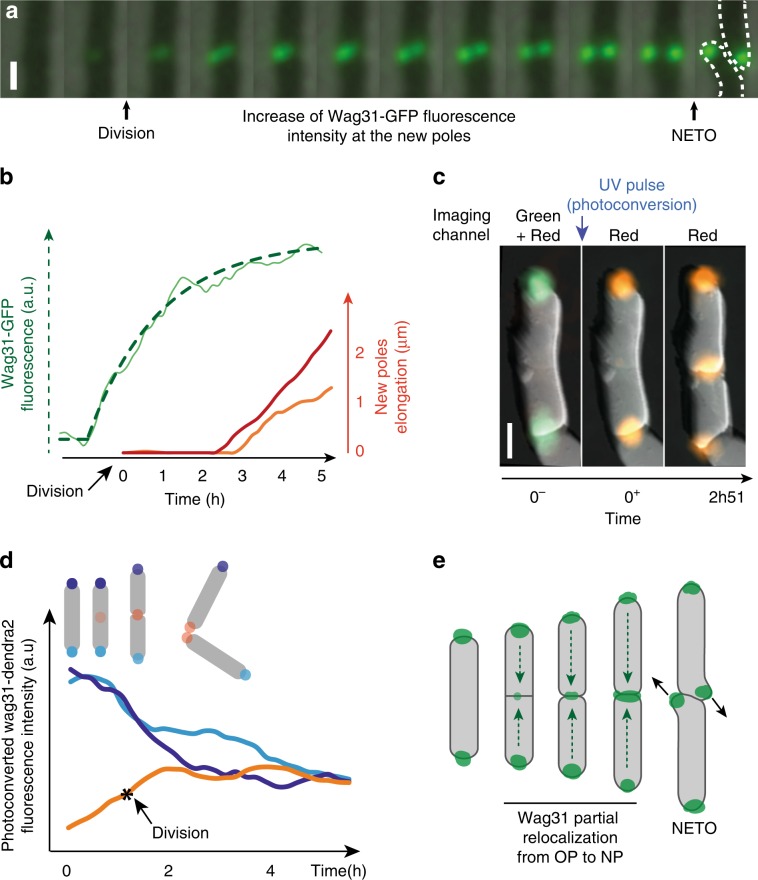
Wag31 relocalizes from the old to the new pole during the pre-NETO phase. **a** Combined time-lapse phase-contrast and fluorescence microscopy of *M. smegmatis* expressing Wag31-GFP. Zoom on the division site showing progressive formation of two elongating new poles. Time between consecutive images is 20 min. Scale bar, 1 μm. **b** New-pole elongation curves of two sibling cells (red and orange) and the average of their Wag31-GFP intensities (green). Data are representative of 20 poles (Supplementary Fig. 7). The *average* intensity rather than *individual* intensity of the two new poles is plotted because their physical proximity precluded individual measurements. Wag31-GFP intensity over time was fitted with an asymptotic exponential function (dotted green line) with a time constant of 1.9 h (confidence interval 0.26 at 95%), adjusted *R*^2^ = 0.98. The confidence interval and *R*^2^ value indicate the quality of the fit to the fluorescence curve. **c** Combined time-lapse fluorescence and atomic force microscopy of *M. smegmatis* expressing Wag31-Dendra2 before (0–), just after (0+), and 2 h 51 min after (2h51) UV-induced photo-conversion. Scale bar, 1 μm. **d** Evolution of photo-converted Wag31-Dendra2 signal at the new and old cell poles during cell division. Each sibling cell has a new pole (orange) and an old pole (light or dark blue) inherited from the mother cell. **e** Schematic representation of Wag31 relocalization from the old pole to the new pole during and after cell division.

Since the pre-NETO phase is not due to physical constraints on the new cell poles, we investigated whether recruitment of the machinery required for cell wall biogenesis might occur with a delay similar to the duration of the pre-NETO phase. We focused on the Wag31 protein (also known as DivIVa) because it preferentially binds to the cell poles^10,30,31^ and functions as a scaffold for recruitment of enzymes responsible for cell wall biogenesis^10^. We measured the progressive accumulation of Wag31 at new cell poles using a strain of *M. smegmatis* expressing a fusion of Wag31 to green fluorescent protein (Wag31-GFP)^7^. Starting from a low level at the time of cell division (about 10% of the final intensity), Wag31-GFP fluorescence at the new cell poles gradually increases before stabilizing, with an average time constant of 2.4 h (representative curve: Fig. 5a; average of 20 poles: Supplementary Fig. 7; Supplementary Movie 2). On average, Wag31-GFP accumulates to about 70% of its final value before NETO occurs (Fig. 5b; Supplementary Fig. 7).

### Wag31 relocalizes from old poles to new poles

Accumulation of Wag31 at the new cell pole during the pre-NETO phase could be due to localization of newly synthesized protein, relocalization of existing protein from the old pole to the new pole, or both. To distinguish between these possibilities, we constructed a strain of *M. smegmatis* expressing a fusion of the Wag31 protein to the photo-convertible fluorescent protein Dendra2 (Wag31-Dendra2). We imaged the cells before and after exposure to a pulse of UV light, which irreversibly converts Dendra2 fluorescence emission from green to red^32^ (Supplementary Fig. 8a). Cells born after photo-conversion showed a gradual accumulation of photo-converted (red) Wag31-Dendra2 at the new poles, which was accompanied by a gradual decrease of photo-converted Wag31-Dendra2 at the old poles (Fig. 5c, d). These observations suggest that Wag31 partially relocalizes from the old pole to the new pole in newborn cells. In parallel, non-photo-converted (green) Wag31-Dendra2 gradually accumulated at both the new and old cell poles, presumably due in part to recruitment of newly synthesized protein (Fig. 5c, d). As a consequence of these dynamics, the total amount of Wag31-Dendra2 increased over time at new cell poles while remaining more or less constant at old cell poles. In subsequent generations, photo-converted Wag31-Dendra2 continued to redistribute between the old and new cell poles (Supplementary Movie 3). Non-dividing cells exhibited a relatively stable signal of photo-converted Wag31-Dendra2 over time, which confirms that the amount of photo-bleaching was low. In contrast, dividing cell lineages exhibited a lower photo-converted Wag31-Dendra2 signal per pole due to repartition of photo-converted Wag31-Dendra2 between a larger number of poles (Supplementary Fig. 8b, c). We conclude that Wag31 partially relocalizes from the old pole to the new pole during the pre-NETO phase (Fig. 5e).

### NETO timing does not scale with the interdivision time

In *M. smegmatis*, cell division and NETO are independent events characterized by different time intervals, although on average the pre-NETO time is only slightly shorter than the interdivision time (Fig. 2). We asked whether NETO dynamics are also characteristic of other mycobacterial species and whether the pre-NETO time scales with the interdivision time. Due to biosafety constraints, we were unable to use AFM to measure single-cell growth in pathogenic mycobacteria. As discussed above, we used AFM-based imaging of *M. smegmatis* to develop a differential image processing method to interpret diffraction-limited optical time-lapse images with improved accuracy (Supplementary Fig. 5). We used this method to measure polar growth of pathogenic mycobacteria with optical time-lapse microscopy.

**Fig. 6:**
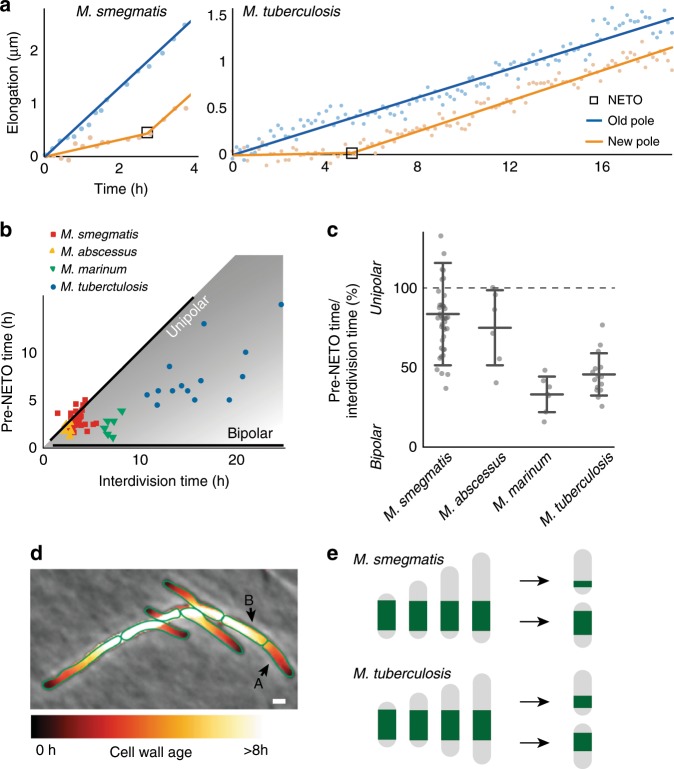
Species-specific NETO dynamics impart different patterns of asymmetry. **a** Elongation dynamics of representative old and new cell poles of *M. smegmatis* and *M. tuberculosis* between birth and division. Points: phase-contrast microscopy data. Lines: linear fit (blue) or bilinear fit (orange). **b** Pre-NETO phase vs. interdivision time in *M. smegmatis* (38 poles), *M. tuberculosis* (14 poles)*, M. abscessus* (6 poles), and *M. marinum* (6 poles). Each symbol represents a new cell pole. Black lines indicate the expected pre-NETO time and interdivision time corresponding to unipolar growth or bipolar growth. **c** Asymmetry of old pole and new pole growth between birth and division in individual cells of *M. smegmatis*, *M. abscessus*, *M. marinum*, and *M. tuberculosis*, expressed as the ratio (pre-NETO time)/(interdivision time). Bars indicate averages and standard deviations. **d** Calculated spatial pattern of cell wall age for individual cells within a microcolony of *M. smegmatis* based on time-lapse phase-contrast microscopy. **e** Calculation of the partitioning of cell wall material inherited by the daughter cells from the original mother cell in *M. smegmatis* (top) and *M. tuberculosis* (bottom), based on the average values calculated from the experimental data in **c** and assuming symmetric division for simplicity.

The interdivision time of the human pathogen *M. tuberculosis* is, on average, about six times longer than the interdivision time of *M. smegmatis*^7,33^. Similar to *M. smegmatis*, we found that new pole growth in *M. tuberculosis* displays NETO growth dynamics, with an initial slow growth phase of variable duration followed by a fast growth phase (Fig. 6a). We also observed NETO dynamics (Supplementary Fig. 10) in *M. abscessus* and *M. marinum*. In *M. smegmatis*, most poles (70%) initiate NETO during the second half of the cell cycle (Fig. 6c). In a small minority of poles (10%), NETO occurs early, in the first half of the cell cycle. Since the pre-NETO time is only slightly smaller on average than the interdivision time in *M. smegmatis*, NETO occurs after division for a minority of cells (about 20%), when the pole is no longer “new”. In the slow-growing mycobacteria *M. marinum* and *M. tuberculosis*, the pre-NETO time is much smaller than the interdivision time, and NETO always occurs before division (Fig. 6b, c).

Unexpectedly, the duration of the lag phase between birth and NETO is only twice as long in *M. tuberculosis* compared to *M. smegmatis*: on average, 6.2 ± 2.2 h in a population of cells growing with an average interdivision time of 16.3 ± 3.1 h (Supplementary Fig. 10). As a consequence, *M. smegmatis* exhibits asymmetric polar growth during 80% of the cell cycle on average, whereas *M. tuberculosis* grows asymmetrically during only 40% of the cell cycle on average (Fig. 6c). Similarly, NETO occurs in the second half of the cell cycle (60% on average) in fast-growing *M. abscessus*, and in the first half of the cell cycle (30% on average) in slow-growing *M. marinum* (Fig. 6c). We conclude that the lag phase between pole birth and NETO does not scale strictly with the interdivision time in mycobacteria (Fig. 6b, c; Supplementary Fig. 9; Supplementary Fig. 10), and the ratio between the pre-NETO time and the interdivision time is variable and determines the degree of growth asymmetry in different species.

Bacterial cells are composed of a combination of newly synthesized material and material inherited from previous generations. We calculated the spatial distribution of aged cell wall material within a cell, and its progression during growth of a microcolony of *M. smegmatis*, based on time-lapse phase-contrast microscopy. Figure 6d shows a microcolony with the cell surface false-colored based on the age of the cell wall (see Methods for details). We found that the composition of aged cell wall material is heterogeneous within the population: some cells are composed mostly of new cell wall material (e.g., cell A in Fig. 6d), while other cells are composed mostly of older material (e.g., cell B in Fig. 6d). Based on NETO growth dynamics measured in *M. smegmatis* and *M. tuberculosis* (Fig. 6c) and assuming symmetric division for simplicity, we calculated the repartitioning of aging cell wall material in the two species. Based on our calculations, we predict that sibling cells of *M. smegmatis* exhibit greater cell-to-cell heterogeneity in the composition of aged cell wall material compared to *M. tuberculosis* sibling cells (Fig. 6e). We conclude that the ratio between the pre-NETO time and the interdivision time affects the cell-to-cell heterogeneity of cell wall age distribution.

## Discussion

Two models of single-cell growth have previously been proposed in mycobacteria: a unipolar model, in which the new cell pole grows slowly (or not at all) between birth and division, and a bipolar model in which both poles (old and new) grow at the same rate after birth^5^. By measuring the growth of each cell pole with unprecedented precision using time-lapse AFM, we find that single-cell growth is neither strictly unipolar nor bipolar; rather, we find that new pole growth follows biphasic NETO dynamics, in which the new pole initiates fast growth after a delay.

In a recent study, Botella et al. used pulse-labeling with fluorescent peptidoglycan precursors and snapshot microscopy to measure the amount of peptidoglycan synthesis at cell poles as a function of cell length, on the assumption that cell length corresponds roughly with cell cycle position^11^. In *M. smegmatis*, they reported a fast rate of peptidoglycan synthesis at one of the cell poles and a slow rate of synthesis at the other cell pole across the whole range of cell lengths. In contrast, they reported that, in *M. tuberculosis*, the rate of peptidoglycan synthesis is lower at one of the cell poles for short cells but is similar at both poles for long cells. Based on this analysis, they proposed that *M. smegmatis* and *M. tuberculosis* exhibit different modes of pole elongation, as reflected in their distinct spatiotemporal dynamics of cell wall peptidoglycan synthesis, although they did not measure cell growth *per se*, nor could they distinguish between old and new cell poles^11^. Our results using time-lapse AFM and optical microscopy to measure cell growth directly are partly consistent with their findings. In *M. tuberculosis*, smaller cells are more likely to be at an earlier time point in the cell division cycle, and therefore the new pole is more likely to be in the pre-NETO phase and to grow slowly. In *M. smegmatis*, NETO occurs, on average, only shortly before division; thus, in the majority of cells one pole (the old pole) grows rapidly for the entire duration of the cell division cycle, while the other pole (the new pole) grows rapidly for only a fraction of the cell cycle just before division. However, we found that NETO in *M. smegmatis* can, in rare cases, occur long before division or, conversely, after division. These results establish that NETO and division are temporally distinct events in both *M. smegmatis* and *M. tuberculosis*. Comparing the dynamics of pole growth in fast-growing and slow-growing *Mycobacterium* species, we conclude that the dynamics of pole growth are fundamentally the same and we propose a unifying model based on our observation that all of the four species we tested display NETO pole dynamics with a pre-NETO lag phase of variable duration.

Biphasic NETO dynamics seem to be a general characteristic of single-cell growth in mycobacteria. The question arises, why do mycobacteria initiate growth of a newly formed pole only after a lag phase? One possibility is that the lag phase corresponds to a period required for “maturation” of the new pole before it can initiate growth^11^. It is, however, unlikely that the maturation phase is an absolute requirement, as initiation of growth from a newly formed pole has been observed immediately after division in a morphologically abnormal strain of *M. smegmatis*^10^. It has been proposed that asymmetric growth has evolved by natural selection because it increases cell-to-cell phenotypic heterogeneity, which may increase overall evolutionary fitness^34^. Consistent with this idea, it was recently shown that deletion of the *lamA* gene in *M. smegmatis* decreases the asymmetry of polar growth as well as survival during exposure to the antibiotic rifampicin^9^. However, we find that polar growth is less asymmetric in slow-growing species (e.g., *M. tuberculosis*) compared to fast-growing species (e.g., *M. smegmatis*), because the pre-NETO time does not scale in proportion to the duration of the cell cycle.

What is the mechanistic basis for NETO pole growth dynamics? In fission yeast, it has been shown that application of a high external mechanical force on the cell poles can counteract the forces driving pole extension, thereby inducing growth arrest^35^. We considered the possibility that pole-to-pole contact forces remaining after cell division might inhibit mycobacterial new pole extension during the pre-NETO phase. However, we find that physical separation of daughter cells immediately after division of the mother cell, using the AFM cantilever, does not eliminate the lag phase. These results rule out the possibility that pole-to-pole contact forces alone are responsible for NETO growth dynamics in mycobacteria.

In fission yeast, it is thought that NETO might reflect the initiation of repartitioning of Cdc42 from the old pole to the new pole^36,37^, although the actual determinants of elongation speed remain unknown^38^. In mycobacteria, polar insertion of new cell wall material is thought to be directed by Wag31, and it has been proposed that accumulation of Wag31 at the new cell pole may be rate-limiting for pole growth^10,39,40^. Consistent with this idea, we found that Wag31 gradually accumulates at the new cell pole prior to NETO due, in part, to relocalization of Wag31 from the old pole to the new pole. However, it remains to be proven whether Wag31 accumulation above a certain threshold actually serves as a trigger for NETO. In other bacteria, relocalization of proteins from one pole to the other can occur on fast timescales. For example, proteins of the Min system in *E. coli* oscillate from one pole to the other with a periodicity of about 50 seconds^41^, and pole-to-pole equilibration of the Wag31 homolog DivIVa in *B. subtilis* takes only about 5 min^42^. These kinetics are much faster than the rate of Wag31 equilibration between the old and new cell poles in *M. smegmatis*, which is about 3 h on average, as shown here. This striking difference suggests that *M. smegmatis* Wag31 and *B. subtilis* DivIVa might have different intrinsic dissociation constants at the poles, or that species-specific regulatory proteins might control the rate of dissociation from the old pole or association to the new pole, such as PonA1^43^ and LamA^9^ in *M. smegmatis*. It has been shown recently that deleting the *lamA* gene decreases growth asymmetry between the two cell poles and increases recruitment of Wag31 to the division septum (and, therefore, to the future new cell poles)^9^. As LamA localizes to the septum^9^, it might reduce the association constant of Wag31 to the new pole, thereby slowing recruitment of Wag31 from the old pole to the new pole.

This study has revealed an unexpected similarity of pole growth dynamics between mycobacteria and fission yeast, which are separated by billions of years of evolution^44^. It seems unlikely a priori that this striking similarity reflects the conservation of molecular mechanisms that evolved prior to the divergence of these organisms. This raises the intriguing possibility that NETO growth dynamics may reflect underlying biophysical and mechanical constraints associated with polar growth of rod-shaped organisms that divide by binary fission. In the future, it will be interesting to determine whether NETO growth dynamics are a general characteristic of pole-growing microorganisms, which are found in taxonomically diverse phyla including the Actinobacteria, Proteobacteria, and Ascomycota^1,29^.

## Methods

### Bacterial strains and growth conditions

*Mycobacterium smegmatis* mc^2^155 (wild-type), *Mycobacterium tuberculosis* Erdman (wild-type), *Mycobacterium abscessus* DSM2 (wild-type), *Mycobacterium marinum* M (wild-type), and derivative strains were grown in Middlebrook 7H9 liquid medium (Difco) supplemented with 0.5% albumin, 0.2% glucose, 0.085% NaCl, 0.5% glycerol, and 0.05% Tween-80. Cultures were grown at 37 °C (30 °C for *M. marinum*) to mid-exponential phase, corresponding to an optical density at 600 nm (OD600) of ∼0.5. Aliquots were stored in 15% glycerol at −80 °C and thawed at room temperature before use; individual aliquots were used once and discarded. The strain expressing a Wag31-GFP fusion protein has been described previously^7^. The reporter strain expressing a Wag31-Dendra2 fusion protein was constructed using the Gibson assembly NEBuilder HiFi DNA Assembly Master Mix (New England Biolabs) according to the manufacturer’s protocol. The *dendra2* open reading frame, minus the start codon, was PCR-amplified from pGEX6P-1-Dendra2 (a gift from Periklis Pantazis; Addgene plasmid # 82436^45^) using primers dendra2_wo_STARTF (AGT TCA ACC GCG GCA ACA ACA ACA CCC CGG GAA TTA ACC) and dendra2_wo_STARTR (GGC CAT TGC GAA GTC ATT ATT TAC CAC ACC TGG CTG GG) and joined by Gibson assembly to the integrative plasmid pIS220^7^, which was PCR-amplified using primers pIS220_wag_F (ATA ATG ACT TCG CAA TGG CCA AG) and pIS_wag_R (GTT GTT GCC GCG GTT GAA C). The resulting plasmid was confirmed by restriction digestion and DNA sequencing. The Wag31-Dendra2-expressing plasmid was integrated at the chromosomal *attB* site by electroporating a wild-type strain of *M. smegmatis* with 1–2 μg of plasmid DNA and selecting transformants on 7H10 plates containing 50 μg ml^−1^ kanamycin.

### Time-lapse optical microscopy

For time-lapse optical microscopy, bacteria were cultured in a custom-made microfluidic device with a continuous flow of 7H9 medium^6,7^. Bacteria were imaged with a DeltaVision personalDV microscope (Applied Precision) equipped with a 100× oil immersion objective and an environmental chamber maintained at 37 °C (30 °C for *M. marinum*). Images were recorded on phase-contrast and fluorescence channels (490/20-nm excitation filter and 528/38-nm emission filter for GFP, 575/25-nm excitation filter and 632/60-nm emission filter for mCherry) with a CoolSnap HQ2 camera.

### Time-lapse AFM

An aliquot from an exponential phase culture of *M. smegmatis* was pipetted onto a PDMS-coated glass coverslip^25^, mounted in a custom-made coverslip holder with a built-in heating unit^46^, and incubated at 37 °C without agitation for ~15 min to allow attachment. Unattached bacteria were removed by rinsing the coverslip surface with 7H9 before imaging. The sample was maintained at 37 °C during imaging using the coverslip heating holder controlled by a TC200 temperature controller (Thorlabs). AFM images were recorded using a customized Icon AFM (Bruker) that was mounted above an inverted optical microscope. Images were recorded at ≈0.5 Hz line rate using ScanAsyst Fluid cantilevers (Bruker) with a nominal spring constant of 0.7 N m^−1^ in PeakForce quantitative nanomechanical mode (QNM) at an oscillation rate of 1 kHz and a force setpoint <2 nN. Fluorescence images were acquired with an EMCCD iXon Ultra 897 camera (Andor) mounted on an IX81 inverted optical microscope (Olympus) equipped with a 100X oil immersion objective. Illumination was provided by a mercury lamp (U-HGLGPS, Olympus). The AFM was mounted above the inverted microscope and the AFM laser was switched off while acquiring fluorescence images.

### Processing of AFM images

Standard scanning probe software (Gwyddion^47^, Nanoscope Analysis) was used to process AFM images. Images were scaled and aligned using Fiji^48^ and the StackReg plugin^49^. The area of interest was cropped, and a montage was generated from the time-series stacks to show consecutive images side by side. A false color was assigned to the background and to each individual bacterium with Photoshop. 3D renderings were made using Fiji, using the AFM height channel for topography and the AFM error channel as an overlay.

### Photo-conversion of Wag31-Dendra2

Photo-conversion of Wag31-Dendra2 was induced with UV light using the excitation channel of a DAPI filter cube (417–477 nm) with 500 milliseconds of exposure time. Subsequent imaging was performed only in the red channel unless specified otherwise, to avoid additional photo-conversion over time.

### Pole elongation measurement with time-lapse AFM

For obtaining pole elongation curves from AFM data, the distance between the pole and the closest immobile surface structure (fiducial marker) was manually measured using Fiji (Fig. 1c; Supplementary Fig. 2). When the reference was changed from one fiducial marker to another, the distance between the old and the new fiducial markers was added as an offset to subsequent values. As the surface structures are inherited by the daughter cells from the mother cell, elongation of a given pole can be measured over several generations.

### Pole elongation measurement with time-lapse phase microscopy

The timing of sibling cell separation was defined in the phase-contrast microscopy data by identifying the abrupt (frame-to-frame) displacement of the two sibling cells along their longitudinal axes (Supplementary Fig. 5). StackReg^49^ was used to align individual bacteria over time using cell shape. Pole elongation was measured with a similar method as with AFM data, using details in cell shape as a fiducial marker instead of the surface nanostructures resolved by AFM (Supplementary Fig. 5).

### Bilinear fitting of pole elongation data

Pole elongation data were fitted with a bilinear curve with a custom MATLAB (R2018a) script, using the MATLAB *fminsearch()* function with four fitting parameters: the timing of the NETO event, the growth speed before NETO, the growth speed after NETO, and an offset.

### Calculation of cell wall age distribution in a microcolony

To compute the cell wall age distribution, we chose a microcolony of bacteria that did not move relative to each other over time. A threshold was applied to separate the cells from the background with Fiji^48^. The binary images were summed to obtain a 2D intensity map indicating the cell wall age distribution for each bacterium within the microcolony.

### Reporting Summary

Further information on research design is available in the Nature Research Reporting Summary linked to this article.

## Supplementary information

Supplementary Information

Description of Additional Supplementary Files

Supplementary Movie 1

Supplementary Movie 2

Supplementary Movie 3

Reporting Summary

## Data Availability

Source data underlying Figs. 2d and 5b-c, and Supplementary Figs. 3, 6, 8, and 11, are provided as a Source Data file. Other data supporting the findings of this study are available from the corresponding authors upon request.

## Source Data

Source Data
