# Supplementary material for: A biphasic growth model for cell pole elongation in mycobacteria
Source: Nat Commun. 2020 Jan 23;11:452. doi: 10.1038/s41467-019-14088-z (PMC6978421; doi:10.1038/s41467-019-14088-z)
Supplement: Supplementary file 1 — Supplementary Information [file 41467_2019_14088_MOESM1_ESM.pdf]

A biphasic growth model for cell pole elongation in mycobacteria

Hannebelle et al.

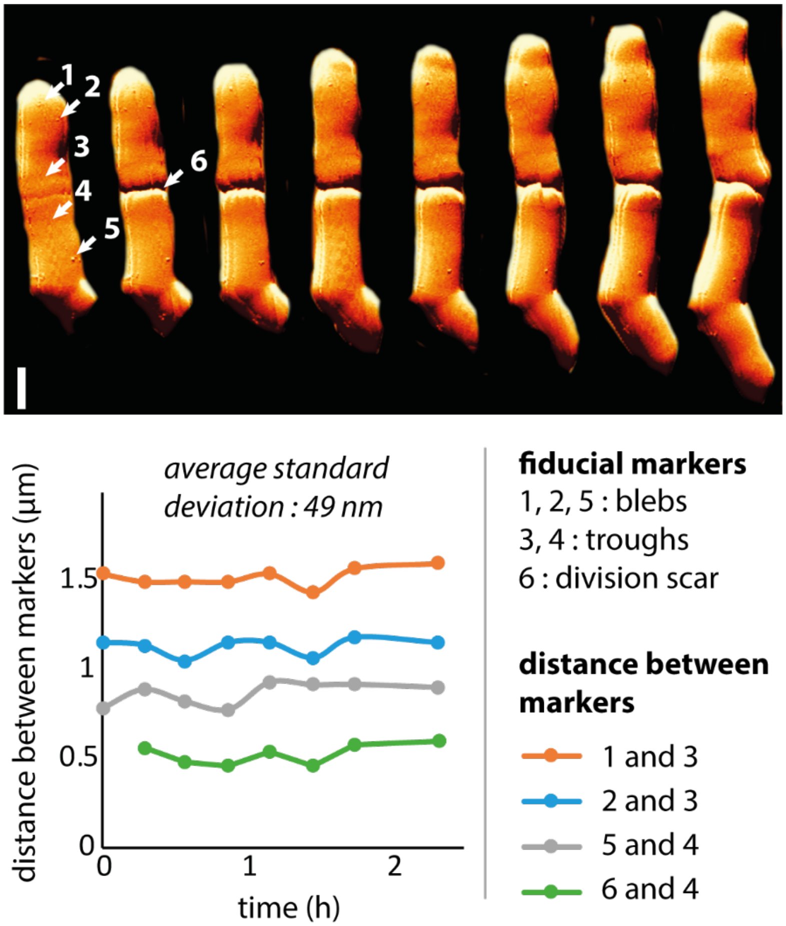

Supplementary Figure 1 | Surface nanostructures resolved by AFM can be used as fiducial markers.

Top panel: AFM time-lapse images of a mother cell and its two daughters. Arrows point to surface nanostructures: blebs (markers 1, 2, 3), wave-troughs (markers 3, 4), and a division scar (marker 6). Scale bar, 1 µm. Bottom panel: Distance between several cell-surface markers and an arbitrarily chosen marker on the same cell (point 3 for the top cell, point 4 for the bottom cell). The distance between markers on the same cell remains constant over time (average standard deviation: 49 nm).

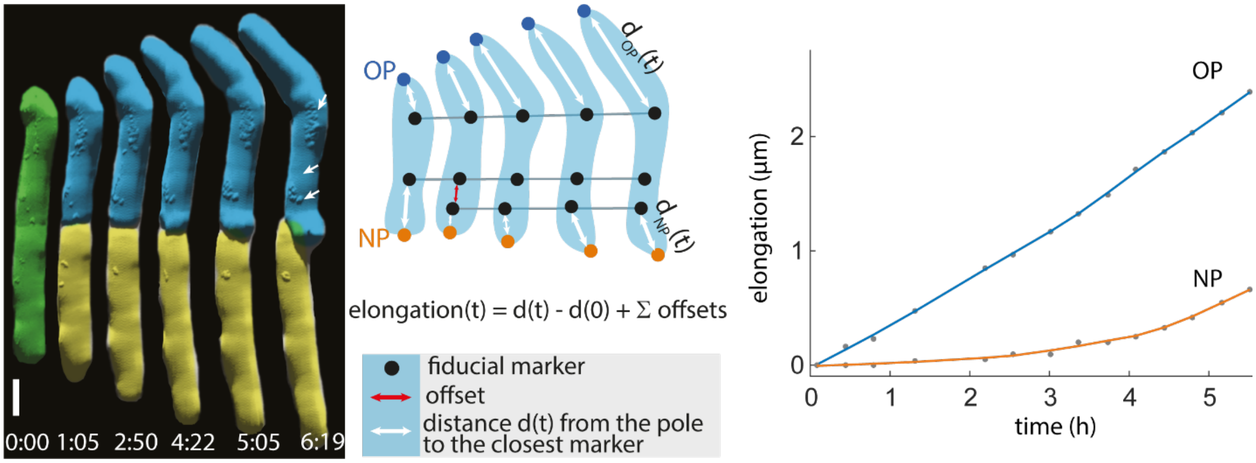

Supplementary Figure 2 | Absolute measurement of pole elongation with AFM using surface nanostructures as fiducial markers.

Left panel: AFM time-lapse images of a mother cell (green) and its two daughters (blue and yellow). Scale bar, 1 µm. Center panel: Schematic representation of the methodology used for measuring pole elongation over time using surface nanostructures as fiducial markers. The elongation of a given pole d(t) is equal to the distance between the pole and the closest fiducial marker (white arrows), plus the cumulated offsets (red arrow) to compensate for switching between different fiducial markers when necessary, minus the initial distance between the pole and the closest marker. Right panel: Corresponding elongation curves for the old pole (OP) and the new pole (NP).

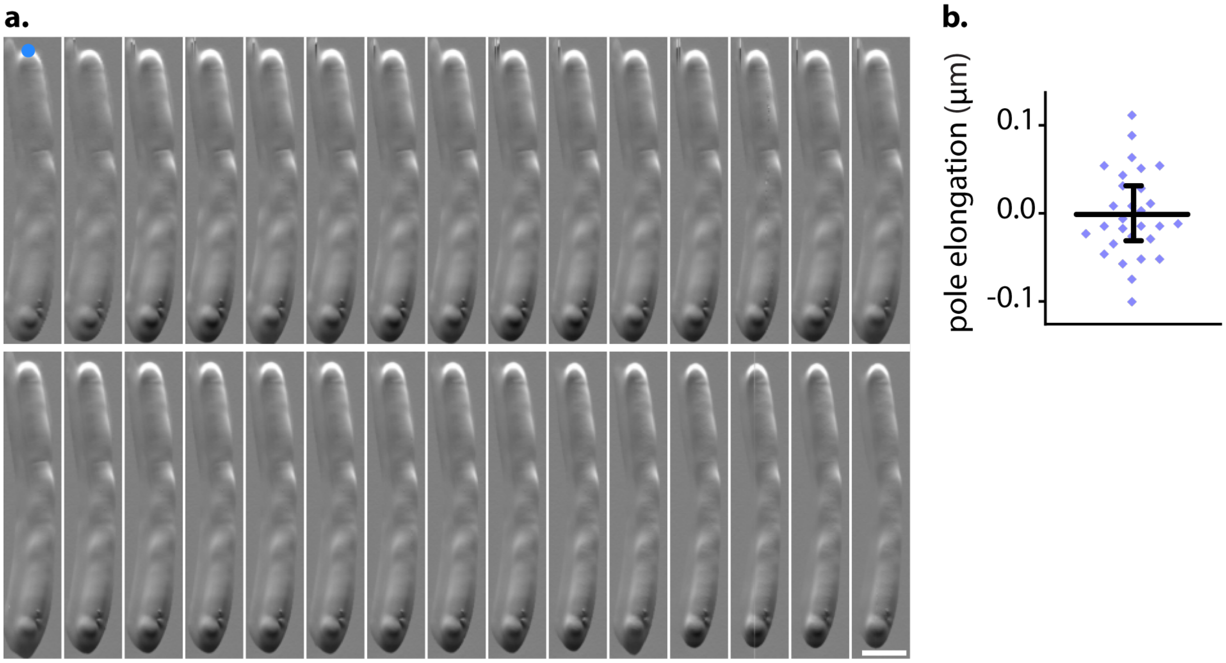

Supplementary Figure 3 | Precision of single-cell measurements using AFM.

(**a**) AFM time-lapse images of a non-growing cell over 15 hours. Blue point: Cell pole used for the measurement. Scale bar, 1 μm. (**b**) Distribution of the measured pole elongation. Each blue point corresponds to a time point in (a). Bars indicate the average and the standard deviation. Standard deviation: 49 nm.

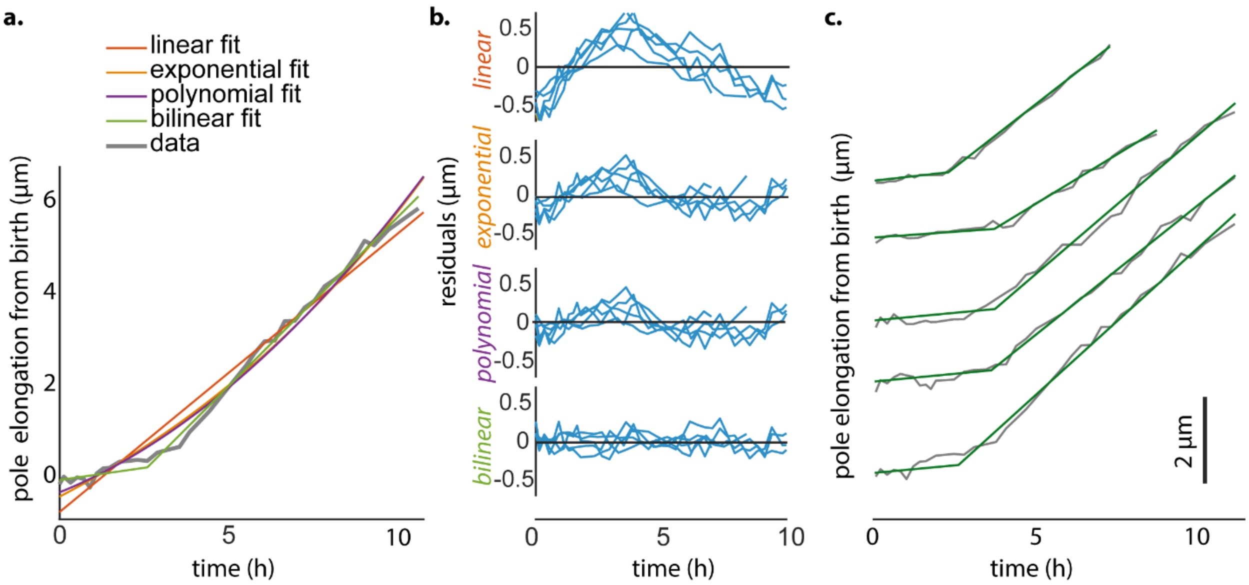

Supplementary Figure 4 | Model for pole elongation dynamics.

(**a**) Elongation of a pole measured from pole birth (grey) fitted with a linear model (red, equation: $y=a \cdot x+b$), an exponential model (orange, $y=a+b \cdot exp(c.x)$), a polynomial model (purple, $y=a \cdot x^2+b \cdot x+c$), or a bilinear model (green). (**b**) Residuals (difference between data and fit) for the four fitting models introduced in (a) and for the elongation of five representative cell poles measured with AFM from pole birth over at least seven hours. Residuals that are systematically positive or systematically negative for much of the data range indicate that the model is a poor fit to the data. Residuals that appear randomly scattered around zero indicate that the model describes the data well[1]. (**c**) Pole elongation of five representative poles measured with AFM (grey), fitted with a bilinear model (green).

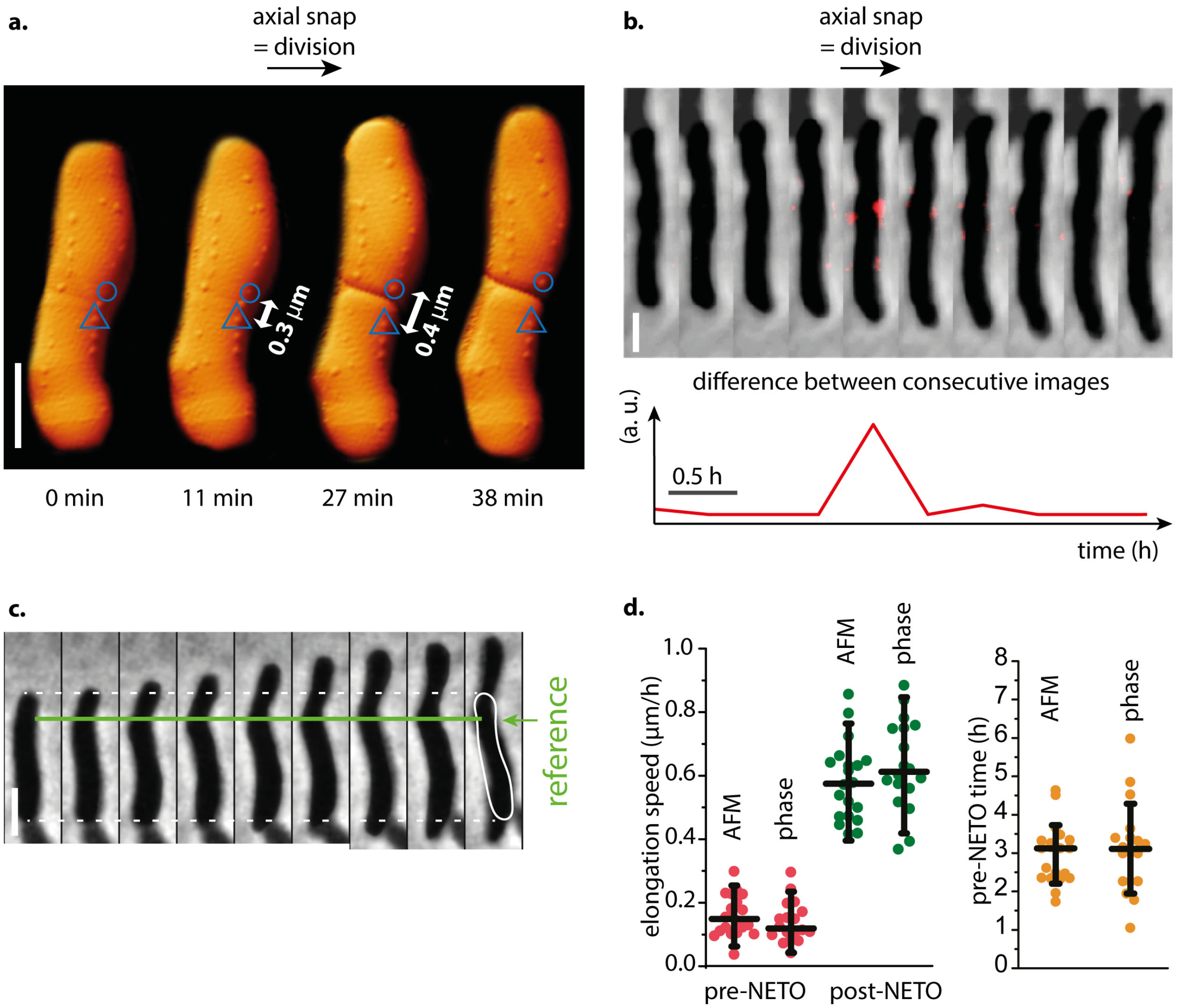

Supplementary Figure 5 | Measurement of pole elongation by phase-contrast microscopy requires a fiducial marker and accurate detection of cell division.

(**a**) Time-lapse AFM illustrating cell division. Blue shapes indicate two surface nanostructures, one inherited by each sibling cell, used as fiducial markers to reveal the axial snap during division. Scale bar, 1 μm. (**b**) Time-lapse phase-contrast microscopy (top panel) and corresponding graph (bottom panel) of the differences between consecutive images (red), revealing the axial snap associated with division. Scale bar, 1 μm. (**c**) Time-lapse phase-contrast microscopy of a single cell from birth (first image) to division (last image). Images were aligned using a kink in cell shape as a fiducial marker (green line), which was used to measure absolute pole elongation over time as for AFM data in Figure 1. Division was detected using the axial snap, as defined in panel (b). Scale bar, 1 μm. (**d**)

Elongation speed before and after NETO and duration of the pre-NETO phase measured with AFM (20 poles) or phase-contrast microscopy (18 poles). Bars indicate average values and standard deviations. The differences between AFM and phase-contrast microscopy measurements are not statistically significant (two-sided t-test; at 0.05 level, when equal variance is not assumed, the difference is not statistically different from 0).

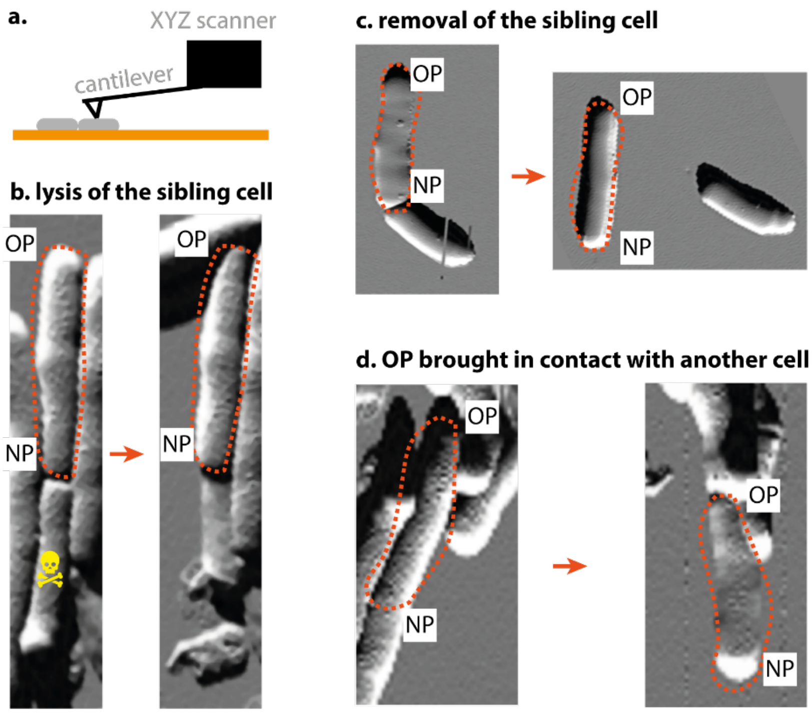

Supplementary Figure 6 | The AFM cantilever tip can be used to lyse or displace cells. (**a**) Schematic of the AFM cantilever applying a force to a mycobacterial cell surface and demonstrating the use of an AFM cantilever as a nano-mechanical manipulating tool. (**b-d**) AFM images, error channel. The observed cell is highlighted with a dotted orange line. (**b**) Lysis of one of the sibling cells (marked with a skull). See Figure 4b. (**c**) Displacement of one sibling cell. See Figure 4c. (**d**) Inversion of the orientation of one of the sibling cells in relation to its neighbors. The old pole (OP) is brought into contact with other cells while the new pole (NP) is removed from contact with other cells. See Figure 4d.

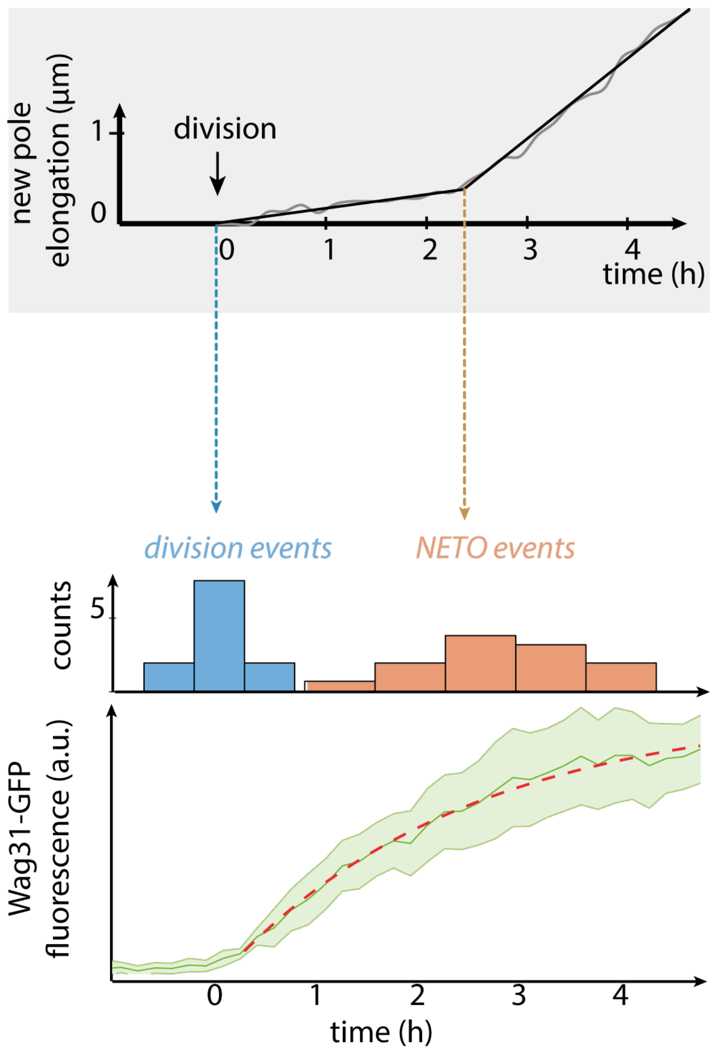

Supplementary Figure 7 | NETO is associated with an accumulation of Wag31-GFP at the new pole.

Elongation measured by time-lapse phase-contrast and fluorescence microscopy of *M. smegmatis*. Top panel: Representative example of new pole elongation dynamics. Middle panel: Binned cell division events and NETO events, extracted from pole elongation curves (20 poles). Bottom panel: Median and standard deviation of the accumulation of Wag31-GFP over time at new poles (20 poles). Dotted red line is an exponential fit of the median values. Time constant (τ) = 2.4 hours. Adjusted R^2 = 0.99.

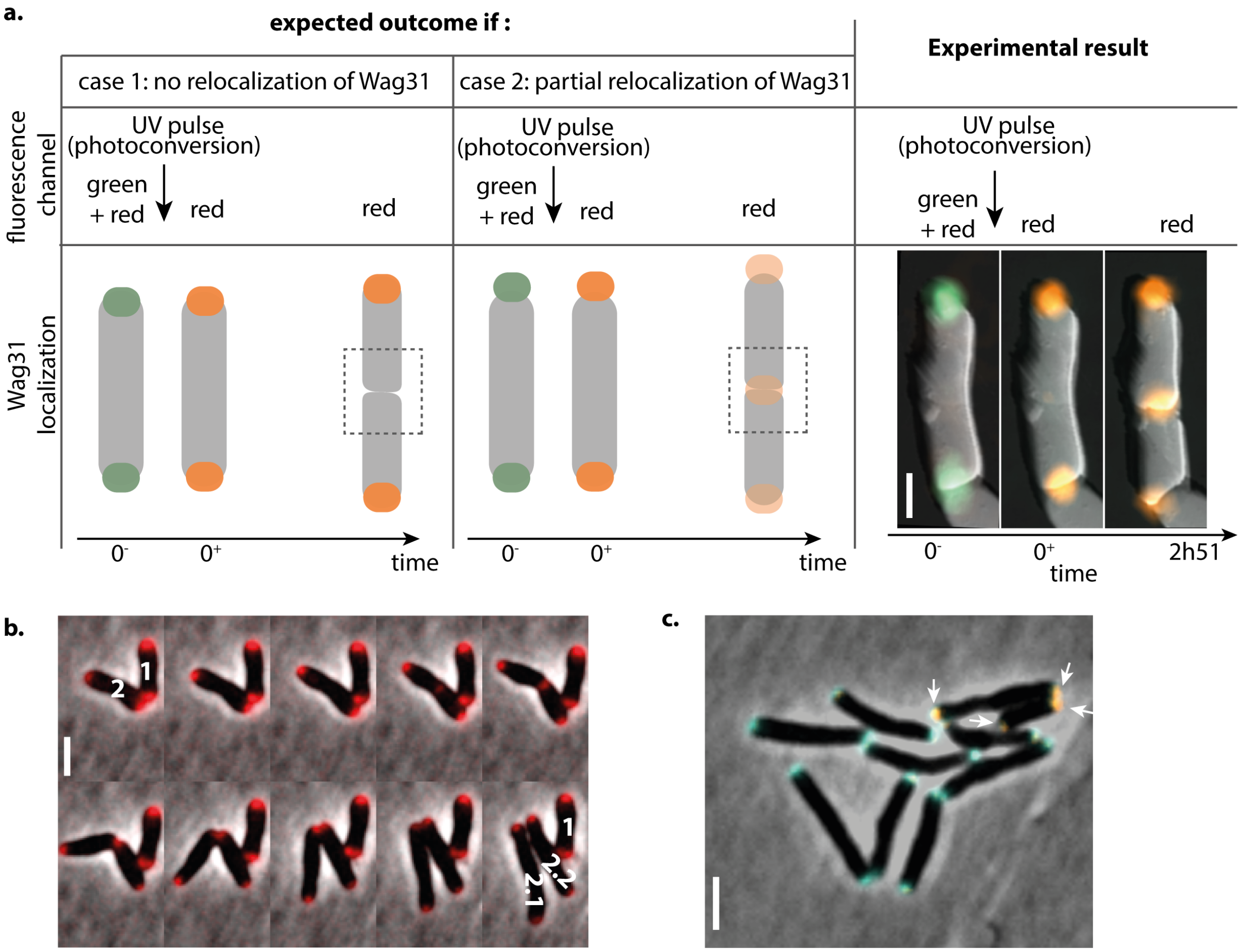

Supplementary Figure 8 | Relocalization of Wag31-Dendra2 from old to new poles measured using photo-conversion fluorescence microscopy.

(**a**) Schematic of the expected experimental results if Wag31 is initially present at the old pole and remains there (case 1), or if Wag31 relocates from the old to the new pole (case 2). Comparison with the experimental results obtained by correlated fluorescence and atomic force microscopy (see Figure 5c). Scale bar, 1 μm. (**b**) Combined phase-contrast and fluorescence time-lapse imaging of photo-converted Wag31-Dendra2 (red) in dividing (cell 2) and non-dividing (cell 1) sibling cells. The photo-converted signal remains relatively stable over time in the non-dividing sibling, suggesting that the gradual decrease of the photo-converted signal in the dividing sibling is due to dilution rather than photo-bleaching. Scale bar, 2 μm. (**c**) Snapshot of combined phase-contrast and fluorescence microscopy from a time-lapse series. The poles marked with white arrows belong to two daughters of a mother cell that grew slowly after photo-conversion. The green poles

belong to eight great-granddaughters of a mother cell that grew normally after photo-conversion. Photo-converted (red) signal at the poles depends on the dilution over the generations and is not necessarily proportional to the non-photo-converted (green) signal. Scale bar, 2 μm.

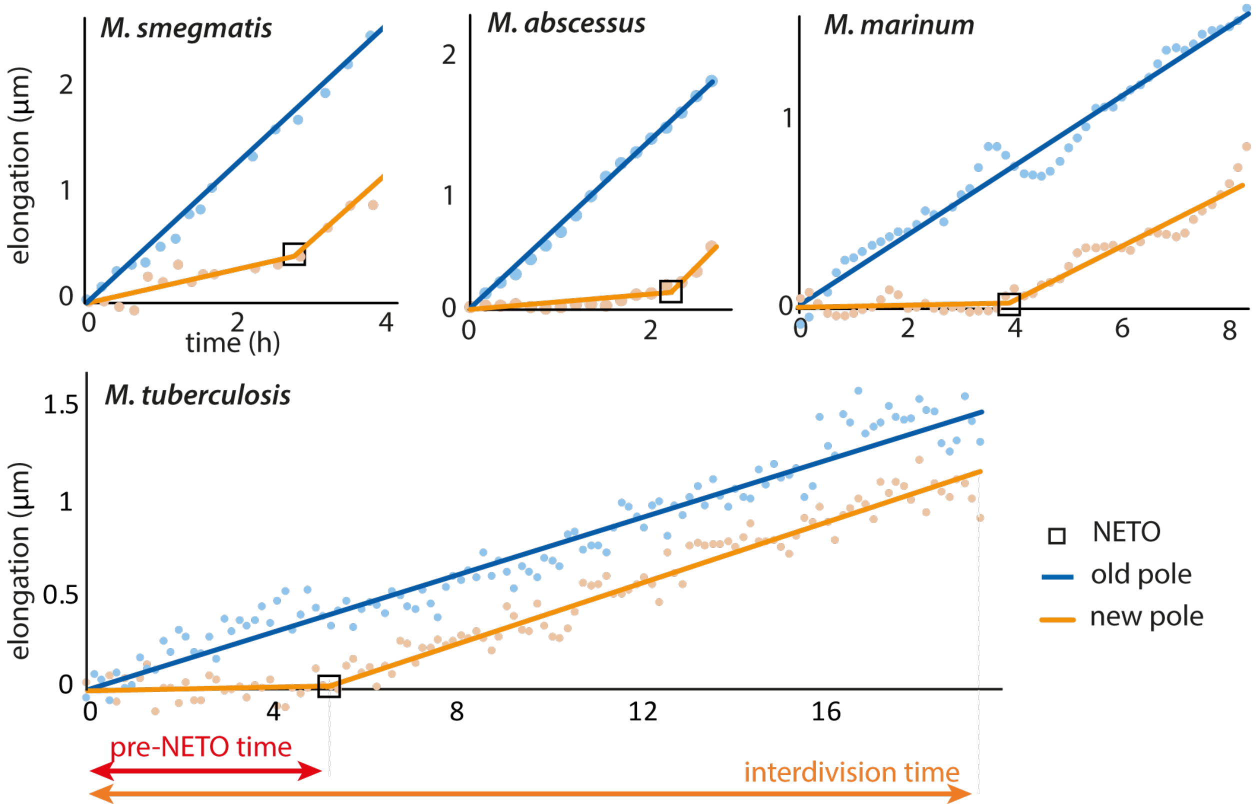

Supplementary Figure 9 | NETO growth dynamics are observed in fast-growing and slow-growing *Mycobacterium* species.

Representative elongation curves for the old pole (blue) and new pole (orange) of a cell between birth and division. NETO (black square) was observed in the four tested species, which include both fast-growers (*M. smegmatis* and *M. abscessus*) and slow-growers (*M. marinum* and *M. tuberculosis*). Points: phase-contrast microscopy data. Blue lines: linear fit. Orange lines: bilinear fit.

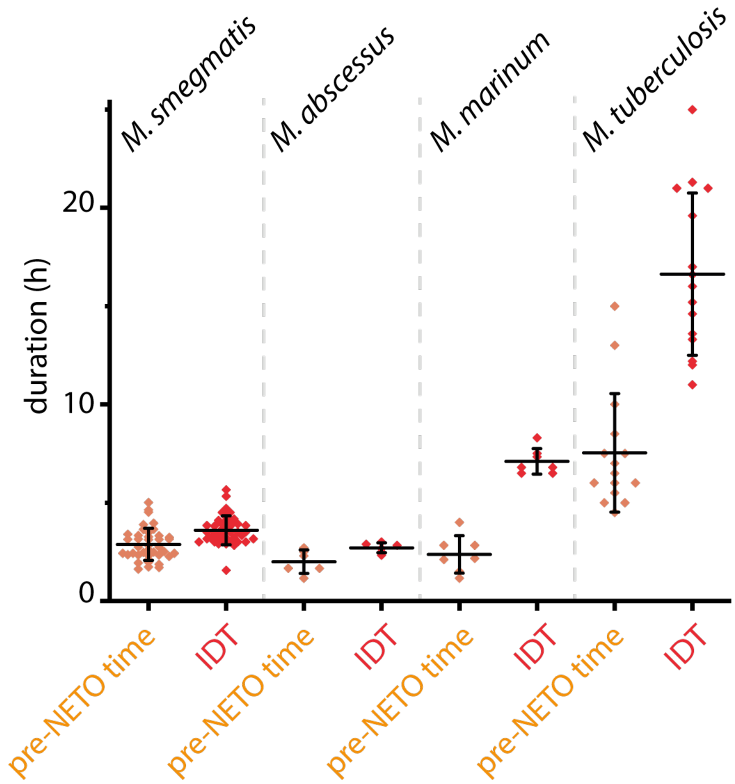

Supplementary Figure 10 | The duration of the pre-NETO phase does not scale in proportion to the interdivision time in *Mycobacterium* species.

Distributions of the duration of the pre-NETO phase and the interdivision time (IDT) for fast-growing (*M. smegmatis* and *M. abscessus*) and slow-growing (*M. marinum* and *M. tuberculosis*) mycobacteria. Each point corresponds to a single pole. Bars indicate averages and standard deviations.

Supplementary References

1. Straume, M. & Johnson, M. L. Analysis of Residuals: Criteria for determining goodness-of-fit. in *Technometrics* **6**, 87–105 (1992).
2. Aldridge, B. B. *et al.* Asymmetry and aging of mycobacterial cells lead to variable growth and antibiotic susceptibility. *Science (80-.).* **335**, 100–104 (2012).
3. Santi, I., Dhar, N., Bousbaine, D., Wakamoto, Y. & McKinney, J. D. Single-cell dynamics of the chromosome replication and cell division cycles in mycobacteria. *Nat. Commun.* **4**, 2470 (2013).
