# Supplementary material for: A biphasic growth model for cell pole elongation in mycobacteria
Source: Nat Commun. 2020 Jan 23;11:452. doi: 10.1038/s41467-019-14088-z (PMC6978421; doi:10.1038/s41467-019-14088-z)
Supplement: Supplementary file 2 — Description of Additional Supplementary Files [file 41467_2019_14088_MOESM2_ESM.pdf]

Description of Additional Supplementary Files

File Name: Supplementary Movie 1
Description: **Time-lapse atomic force microscopy (AFM) of *M. smegmatis* growth and division.** Time-lapse AFM height (3D) movie with the error channel overlaid as a skin reveals pole elongation and the spatial localization of nanostructures apparent at the cell surface.

File Name: Supplementary Movie 2
Description: **Correlated phase-contrast and fluorescence time-lapse microscopy of *M. smegmatis* expressing Wag31-GFP.** Scale bar, 5 µm.

File Name: Supplementary Movie 3
Description: **Correlated phase-contrast and fluorescence time-lapse microscopy of *M. smegmatis* expressing Wag31-Dendra2 before (green) and after (red) UV-induced photo-conversion.** Scale bar, 10 µm.
