# Supplementary material for: A biphasic growth model for cell pole elongation in mycobacteria
Source: Nat Commun. 2020 Jan 23;11:452. doi: 10.1038/s41467-019-14088-z (PMC6978421; doi:10.1038/s41467-019-14088-z)
Supplement: Supplementary file 6 — Reporting Summary [file 41467_2019_14088_MOESM6_ESM.pdf]

nature research

Corresponding author(s): Georg E. Fantner, John D. McKinney

Last updated by author(s): Apr 17, 2019

Reporting Summary

Nature Research wishes to improve the reproducibility of the work that we publish. This form provides structure for consistency and transparency in reporting. For further information on Nature Research policies, seeAuthors & Referees and theEditorial Policy Checklist .

Statistics

For all statistical analyses, confirm that the following items are present in the figure legend, table legend, main text, or Methods section.

n/a	Confirmed	
☐	☒	The exact sample size (*n*) for each experimental group/condition, given as a discrete number and unit of measurement
☐	☒	A statement on whether measurements were taken from distinct samples or whether the same sample was measured repeatedly
☐	☒	The statistical test(s) used AND whether they are one- or two-sided<br>*Only common tests should be described solely by name; describe more complex techniques in the Methods section.*
☐	☒	A description of all covariates tested
☐	☒	A description of any assumptions or corrections, such as tests of normality and adjustment for multiple comparisons
☐	☒	A full description of the statistical parameters including central tendency (e.g. means) or other basic estimates (e.g. regression coefficient) AND variation (e.g. standard deviation) or associated estimates of uncertainty (e.g. confidence intervals)
☐	☒	For null hypothesis testing, the test statistic (e.g. *F*, *t*, *r*) with confidence intervals, effect sizes, degrees of freedom and *P* value noted<br>*Give P values as exact values whenever suitable.*
☐	☒	For Bayesian analysis, information on the choice of priors and Markov chain Monte Carlo settings
☐	☒	For hierarchical and complex designs, identification of the appropriate level for tests and full reporting of outcomes
☐	☒	Estimates of effect sizes (e.g. Cohen's *d*, Pearson's *r*), indicating how they were calculated

Our web collection on statistics for biologists contains articles on many of the points above.

Software and code

Policy information about availability of computer code

Data collection	For data collection, were used:<br>- commercial softwares: Nanoscope v9.4, Andor Solis 4.3, softWoRx
Data analysis	For data analysis, were used:<br>- commercial softwares: Matlab (R2018a), Nanoscope Analysis 1.7<br>- open-source sofwares: Fiji (imageJ 1.52n), gwyddion

For manuscripts utilizing custom algorithms or software that are central to the research but not yet described in published literature, software must be made available to editors/reviewers. We strongly encourage code deposition in a community repository (e.g. GitHub). See the Nature Research guidelines for submitting code & software for further information.

Data

Policy information about availability of data

All manuscripts must include a data availability statement. This statement should provide the following information, where applicable:

- Accession codes, unique identifiers, or web links for publicly available datasets
- A list of figures that have associated raw data
- A description of any restrictions on data availability

The data that support the findings of this study are available from the corresponding author upon request.

Field-specific reporting

Please select the one below that is the best fit for your research. If you are not sure, read the appropriate sections before making your selection.

Life sciences study design

All studies must disclose on these points even when the disclosure is negative.

Sample size	As AFM is a low throughput technique, we used a limited number of M. smegmatis cells. Our statistical analysis was done only once on a dataset of n=20 cell poles. No additional data on cell pole growth were added afterwards to increase this number. We chose to analyse a similar number (n=18) of cell poles by optical microscopy. The similarity between the results we obtained independantly using AFM and optical microscopy make us believe that these sample sizes were sufficient.
Data exclusions	Cells that where not visible during at least a cell cycle were not considered (e.g. cells loosely attached to the surface, and detaching over the course of the AFM imaging experiment). Optical time-lapse movies of low qualities (i.e. out of focus) were removed. Due to the restrictions of our method for measuring polar growth with phase microscopy images, we analyzed only cells with a distinct morphological feature in phase-contrast datasets (e.g. constriction, bent shape).
Replication	When possible (non-pathogenic M. smegmatis), we confirmed our results with both AFM and optical microscopy time-lapses in independant experiments on two different setups (Figure S4, Figure 4&S6-7). This was not possible for<br>1. pathogenic species, for which only optical microscopy was used (Figure 5, Figure S8).<br>2. micromanipulation experiments, where only AFM can be used (Figure 3). In that case, as all three independent experiments show coherent results (i.e. the pre-NETO phase is visible with and without physical contraints on the poles), we are confident that our conclusion remains valid.
Randomization	Does not apply.
Blinding	Does not apply.

Reporting for specific materials, systems and methods

We require information from authors about some types of materials, experimental systems and methods used in many studies. Here, indicate whether each material, system or method listed is relevant to your study. If you are not sure if a list item applies to your research, read the appropriate section before selecting a response.

Materials & experimental systems

n/a	Involved in the study	
☒	☐	Antibodies
☒	☐	Eukaryotic cell lines
☒	☐	Palaeontology
☒	☐	Animals and other organisms
☒	☐	Human research participants
☒	☐	Clinical data

Methods

n/a	Involved in the study	
☒	☐	ChIP-seq
☒	☐	Flow cytometry
☒	☐	MRI-based neuroimaging
